# Inhibition of histone deacetylase in Arabidopsis root calli promotes *de novo* shoot organogenesis

**DOI:** 10.3389/fpls.2024.1500573

**Published:** 2025-01-27

**Authors:** Qinwei Pan, Ruirui Huang, Qiong Xiao, Xuting Wu, Baoxia Jian, Yanan Xiang, Lijun Gan, Zongrang Liu, Yi Li, Tingting Gu, Huawei Liu

**Affiliations:** ^1^ State Key Laboratory of Crop Genetics and Germplasm Enhancement, College of Horticulture, Nanjing Agricultural University, Nanjing, China; ^2^ Laboratory of Plant Hormone, College of Life Sciences, Nanjing Agricultural University, Nanjing, China; ^3^ USDA-ARS, Appalachian Fruit Research Station, Kearneysville, WV, United States; ^4^ Department of Plant Science and Landscape Architecture, University of Connecticut, Storrs, CT, United States; ^5^ State Key Laboratory of Desert and Oasis Ecology, Key Laboratory of Ecological Safety and Sustainable Development in Arid Lands, Xinjiang Institute of Ecology and Geography, Chinese Academy of Sciences, Urumqi, China

**Keywords:** TSA application, histone deacetylase inhibition, callus induction, shoot regeneration, Arabidopsis

## Abstract

*De novo* organogenesis from somatic cells to the entire plant represents a remarkable biological phenomenon, but the underlying regulatory mechanism, particularly at the epigenetic level, remains obscure. In this work, we demonstrate the important role of histone deacetylases (HDACs) in shoot organogenesis. HDAC inhibition by trichostatin A (an HDAC inhibitor) at the callus induction stage promotes shoot formation in wounded roots and circumvents tissue wounding to initiate shoot regeneration in unwounded roots. This HDAC inhibition-mediated promotion of shoot organogenesis in wounded roots is associated with the concomitant upregulation of the wound signaling pathway (*WOUND INDUCED DEDIFFERENTIATION 4, ENHANCER OF SHOOT REGENERATION1, ISOPENTENYLTRANSFERASE 5*, *CUP-SHAPED COTYLEDON 2* etc.) and the ARF-LBD pathway (*AUXIN RESPONSE FACTOR 19, LATERAL ORGAN BOUNDARIES-DOMAIN 29*, etc.) and the downregulation of auxin biosynthesis and reduced auxin content. Furthermore, inhibiting HDACs enhances the local enrichment of histone 3 lysine 9/lysine 14 acetylation at *ISOPENTENYLTRANSFERASE 5*, supporting the role of histone acetylation in its transcriptional regulation. On the other hand, the HDAC inhibition-associated activation of shoot organogenesis from unwounded roots is coupled with increased expression of the *ARF-LBD* pathway gene *LATERAL ORGAN BOUNDARIES-DOMAIN 29* while bypassing the wound signaling or auxin biosynthetic genes. These findings provide novel insights into the regulatory mechanisms underlying *de novo* shoot organogenesis and lay a foundation for the improvement of plant transformation technologies.

## 1 Introduction

*De novo* shoot organogenesis (DNSO) is dictated by the acquisition of pluripotency by somatic cells, which enables multicellular organisms to regenerate organs. DNSO in Arabidopsis can be achieved via a two-step media culture process (Skoog and Miller, 1957; Duclercq et al., 2011). First, wounded explants are cultured in excessive auxin-containing callus-inducing medium (CIM) to induce the formation of pluripotent cell masses, commonly termed calli. Then, shoots are regenerated from callus cells after they are transferred to the shoot-inducing medium (SIM) with a high cytokinin/auxin ratio (Skoog and Miller, 1957; Ikeuchi et al., 2013). Studies using Arabidopsis (*Arabidopsis thaliana*) wounded roots revealed that DNSO is achieved via a lateral root initiation program involving the formation of tissues with root meristem identity from pericycle-like cells (Duclercq et al., 2011). Understanding the molecular mechanisms underlying DNSO is highly important for both fundamental research and plant genetic engineering.

DNSO occurs in response to dramatic changes in cell identity and growth patterns, which are regulated by hormonal homeostasis and external stimuli (Su and Zhang, 2014; Xu and Huang, 2014). Hormone-induced DNSO acts through the ARF-LBD (*AUXIN RESPONSE FACTOR-LATERAL ORGAN BOUNDARIES* domain) pathway, which translates auxin stimuli into regulatory signals to control cell cycle activity and callus formation (Ikeuchi et al., 2019). On the other hand, wound signaling is a prerequisite for shoot organogenesis (Iwase et al., 2015). The physical damage- or wound-induced DNSO process apparently requires *WOUND INDUCED DEDIFFERENTIATION (WIND*) family proteins and *ENHANCER OF SHOOT REGENERATION 1* (*ESR1*), which are distinct from ARF-LBD factors. Ectopic expression of *WIND1* or *ESR1* induces the initiation of shoot regeneration from root segments or hypocotyls in Arabidopsis, whereas loss-of-function *wind1* or *esr1* mutants exhibit reduced callus production, indicating that wounding-induced DNSO is mechanistically distinct from that induced by plant hormones (Banno et al., 2001; Iwase et al., 2011, 2015, 2017). However, the failure of the loss-of-function *wind1* or *esr1* mutant to completely abrogate the callus formation or shoot regeneration process suggests that the two pathways may be functionally complementary. Indeed, tissue wounding induces the expression of both hormone biosynthetic genes and hormone-responsive genes (Ikeuchi et al., 2017), highlighting the common components exploited by both regulatory mechanisms and pathways.

Recent studies have revealed complex molecular interactions among wounding, hormones, transcription factors and histone modifications that regulate pluripotency acquisition and subsequent shoot organogenesis (Xu and Huang, 2014; Lee et al., 2016; Liu et al., 2016; Song et al., 2016; Kim et al., 2018; Lee et al., 2018; Ishihara et al., 2019; Wu et al., 2022). Reprogramming trimethyl-histone H3 lysine 27 (H3K27me3) is critical during the leaf-to-callus transition in Arabidopsis (He et al., 2012). The chromatin landscape, represented by histone modifications and chromatin accessibility, is closely associated with the activation of shoot identity genes during shoot regeneration (Wu et al., 2022). Several genes essential for shoot formation are regulated by histone methyltransferases and demethylases (Li et al., 2011; Liu et al., 2016; Lee et al., 2018). Furthermore, pronounced alterations in histone acetylation occur after wounding in a few genes (*e.g., WIND1*, *ERF113/RAP2.6L* and *LBD16*) that reprogram the wound-inducible organogenesis process (Rymen et al., 2019).

Acetylation of the H3 or H4 histone tail makes chromatin more accessible to transcription factors and other DNA-binding proteins, making the chromatin environment more conducive to gene transcription. The transcriptional activity or gene expression state is influenced by the balanced activity of histone acetyltransferases (HATs) and HDACs (Eberharter and Becker, 2002). In plants, HATs and HDACs regulate cell/callus proliferation and cell differentiation in various cases. Mutation of the gene encoding the histone acetyltransferase *HAG1/AtGCN5* promotes callus formation but not shoot regeneration in Arabidopsis (Kim et al., 2018). Repression or deficiency of HDAC-containing complexes leads to accelerated growth of calli (Furuta et al., 2011; Lee et al., 2016). Treating tissues with trichostatin A (TSA), which inhibits the enzymatic activity of HDACs, induces the formation of an embryo-like structure in true leaves in Arabidopsis (Tanaka et al., 2008). TSA also promotes the induction of microspore-derived embryos in wheat (Jiang et al., 2017), suggesting an essential role of HDACs in embryogenesis. TSA has sometimes been used as an additive for enhancing tissue regeneration/somatic embryogenesis (Bie et al., 2020; Jiang et al., 2017). However, the mechanisms underlying TSA treatment are elusive.

In this study, we demonstrated that in Arabidopsis, the HDAC inhibitor TSA promoted shoot regeneration from wounded roots and circumvented tissue wounding to activate shoot regeneration in unwounded roots. This HDAC inhibition-mediated promotion of shoot regeneration in wounded roots is associated with concomitant increases in wound signaling and *ARF-LBD* pathways and reductions in auxin biosynthesis. The activation of shoot regeneration from unwounded roots is associated with increased *ARF-LBD* pathway activity. In addition, we provide evidence that the upregulation of some shoot formation-associated genes, such as *CUC2* and *IPT5*, by inhibiting HDAC is coupled with increased levels of local histone 3 lysine 9/lysine 14 acetylation (H3K9/K14ac). Our findings highlight the important role of HDACs in shoot regeneration and provide insights into the mechanism underlying the regulation of *de novo* shoot organogenesis.

## 2 Materials and methods

### 2.1 Plant growth and TSA treatment

Seedlings of *Arabidopsis* (*Arabidopsis thaliana*, Columbia-0*)* were cultured on 1/2 MS medium in a growth chamber (16/8 h light conditions at 22 ± 2°C, 6000 lux, 70–80% relative humidity). The two-step media culture process was described as before (Xiang et al., 2018). The roots of the 7-day-old seedlings were cut into 0.5 cm segments and transferred to CIM supplemented with different concentrations of TSA (T1952-200, Sigma). TSA was dissolved in DMSO (20 mM) and added to CIM or SIM at final concentrations of 0, 3 and 15 µM. The CIM was composed of B5 medium supplemented with 2.2 μM 2,4-D and 0.2 μM BA. The SIM contained B5 medium supplemented with 5.0 μM isopentenyladenine and 0.9 μM indole-3-acetic acid (Che et al., 2006). The wounded roots cultured on CIM or SIM were kept in a growth chamber under 24 h light conditions at 22 ± 2°C with 70–80% relative humidity (3500 lux). The formation of green callus foci and regenerated shoots was monitored. At least three biological replicates were performed, with 50 explants per replicate.

Seven-day-old seedlings were used to test the effect of TSA on shoot regeneration from unwounded roots. Seedlings were cultured on CIM with or without 3 μM TSA for 4 days and then transferred to TSA-free SIM. Photographs were taken every 3 days.

### 2.2 Semithin sections

The root segments were collected and fixed immediately in Formal-Acetic-Alcohol (FAA) prepared in the v/v ratio 5:5:50:40 of formaldehyde for 24 h. After fixation, the samples were dehydrated in 75% ethanol, followed by soaking in 100% isopropanol for 10 h and then in 100% 1-butanol for 10 h. The dehydrated tissues were then placed in glycol methacrylate (GMA) for infiltration. After infiltration, the samples were transferred to GMA and allowed to polymerize overnight at 60°C. The sections were stained with 1% toluidine blue for 30 min and subsequent to rinse with distilled water for 3-5 s. Photographs were taken with an Olympus BX43 microscope system and a Canon EOS 700D.

### 2.3 RNA sequencing and data processing

**Figure 1:**
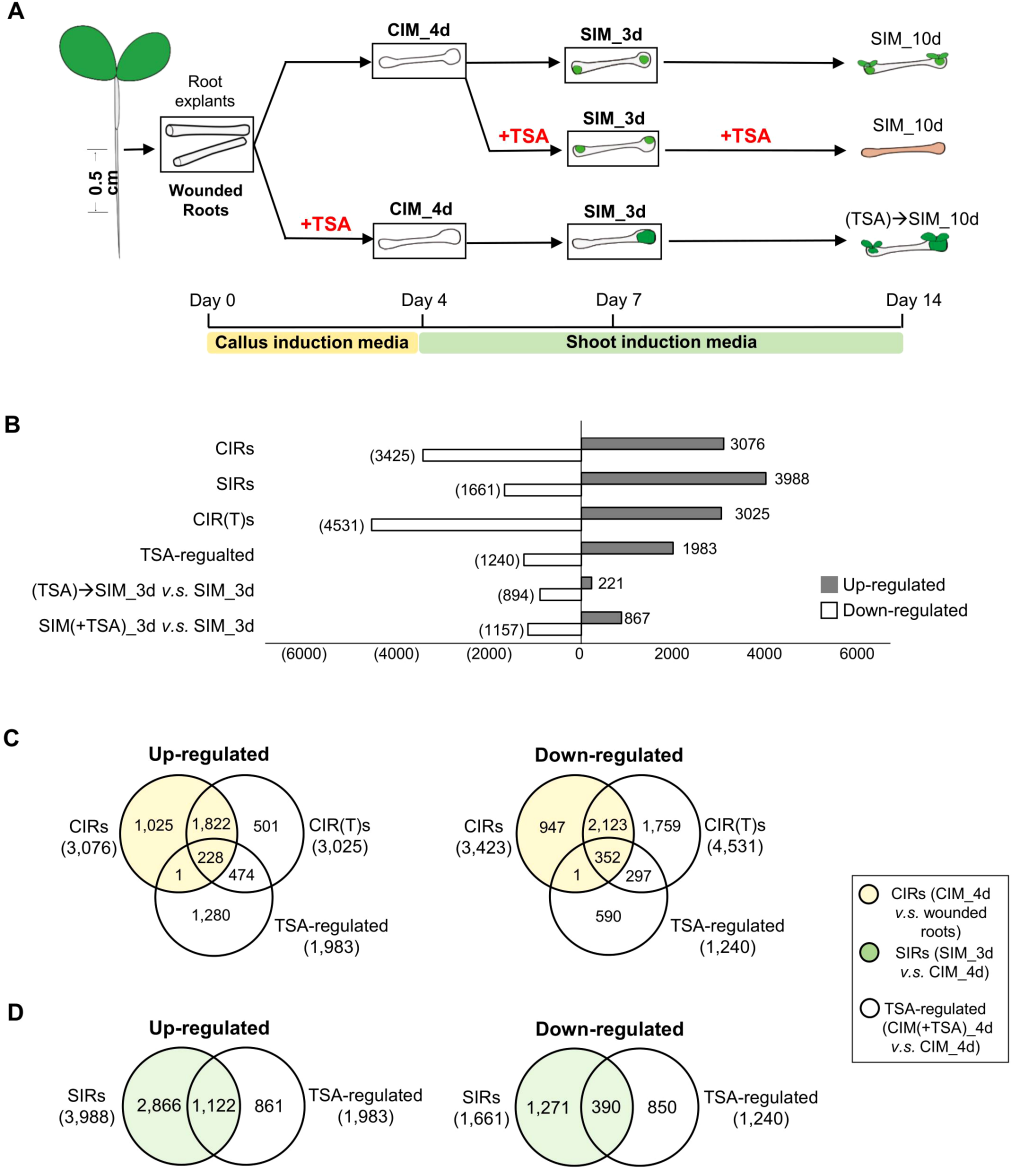
Transcriptome analyses of the effect of TSA on wounded roots. **(A)** An illustration of the cultural conditions and time points for the collection of wounded roots materials for RNA-seq. The tissues collected for RNA-seq are bolded and framed. The samples collected are the root segments cut from seven-day-old seedlings (“wounded roots”), incubated for 4 days on CIM with or without TSA (“CIM_4d” or “CIM(+TSA) 4d”), cultured for 3 days on SIM following a 4-day callus induction with or without TSA in CIM (“SIM_3d” or “(TSA)->SIM_3d”), and treated with TSA for 3 days on SIM following a 4-day incubation on TSA-free CIM (“SIM(+TSA) 3d”). **(B)** DEGs (fold change>2, FDR<0.05) during callus and shoot formation. **(C, D)** Venn diagrams show that TSA in CIM regulates the expression of genes involved in callus formation and shoot regeneration in wounded roots. CIRs, DEGs during callus induction (CIM_4d vs. wounded roots); SIRs, DEGs during shoot induction (SIM_3d vs. CIM_4d); TSA-regulated, DEGs by TSA treatment in CIM (CIM(+TSA) 4d vs. CIM_4d).

For RNA-seq, wounded roots were collected at six time points/treatments (**Figure 1A**): wounded roots collected from 7-day-old seedlings (“wounded roots”), after incubation for 4 days on TSA- or TSA+ CIM (“CIM_4d” or “CIM(+TSA)_4d”), after culturing on SIM for 3 days following a 4-day preincubation on TSA- or TSA+ CIM (“SIM_3d” and “(TSA)→SIM_3d”), and wounded roots cultured on TSA+ SIM for 3 days following a 4-day preincubation on CIM (“SIM(+TSA)_3d”). One wounded roots explant segment was collected from each seedling. Unwounded root materials were collected from the seedlings cultured on CIM for 4 days with or without TSA treatment. Three biological replicates with approximately 150 roots/sample were pooled together to obtain sufficient amounts of tissue for RNA extraction. The final concentration of TSA was adjusted to 3 μM.

RNA extraction, sequencing and data processing were performed as previously described (Gu et al., 2019). Clean reads were aligned to the *TAIR10* genome (https://www.arabidopsis.org/) via HISAT2 v2.1.0 using the default parameters (Kim et al., 2015). The number of fragments per kilobase of transcript sequence per million base pairs sequenced (FPKM) was calculated (Trapnell et al., 2010). Differential expression analyses were performed via the edgeR package (3.24.3). Benjamini and Hochberg’s approach was used to adjust the P values to control the false discovery rate (FDR). Expressed genes (FPKM>0.3) with *P_adj_* < 0.05 and >2-fold change were regarded as differentially expressed genes (DEGs) if not indicated. We verified the expression profiles of 11 DEGs via quantitative real-time PCR, which supported the reliability of our RNA-seq data (**Supplementary Figure S1**). The expression profiles for each gene in the roots explants and the wounded roots are shown in **Supplementary Data 1** and **Data 2**, respectively.

The wound-regulated and *BROWN-MIDRIB* 3 (*BM3*)-regulated genes in the wounded roots were adapted from previously published data (Rymen et al., 2019). For consistency with previously published work (Rymen et al., 2019), the related DEG analyses were based on FC>1.5 and *P_adj_* <0.001.

### 2.4 ChIP-seq experiments and data processing

ChIP-seq experiments for H3K9/K14ac (Millipore, 07-329) were performed in duplicate using wounded roots explant segments cultured on CIM for 4 days with or without TSA treatment. One or two segments were taken from each seedling. The native ChIP protocol (Huang et al., 2020) was used to profile the genome-wide H3K9/K14ac enrichment. Briefly, 1 g of root tissue was ground into a fine powder in liquid nitrogen. The nuclei were isolated in extraction buffer and then digested with micrococcal nuclease for chromatin fragmentation. Library amplification and sequencing were performed at the Beijing Genomics Institute (BGI) as previously described (Huang et al., 2020). The ChIP-seq reads were aligned to the *TAIR10* Arabidopsis genome using Bowtie2 (Langmead and Salzberg, 2012). The mapping reads and consensus narrow peaks of the two replicates were used to calculate the differential acetylation regions using DiffBind (v2.10.0).

### 2.5 Immunoblotting

Proteins were extracted by boiling in SDS sample buffer and electrophoresed on an SDS-PAGE gel. The primary antibodies used were anti-histone H3 (ab1791; Abcam; 1:2000) and anti-acetyl-histone H3 (clone 62-141-13; Millipore; 1:2000). A secondary goat anti-rabbit horseradish peroxidase antibody (Abmart) was used at a 1:2000 dilution, and signals were detected via enhanced chemiluminescence (SuperSignal West Femto Chemiluminescent Substrate; Pierce).

### 2.6 Quantitative real-time PCR analyses

A total of 1 μg of RNA from each sample was used to synthesize first-strand cDNA via M-MLV reverse transcriptase according to the manufacturer’s instructions (TaKaRa, Dalian, China). We performed qRT-PCR on an Applied Biosystems 7500 real-time PCR system using a SYBR Green RT-PCR kit (Novland, Shanghai, China). All the primers used are listed in **Supplementary Table S1**. Three biological and three technical replicates were performed for each gene. Relative expression levels were calculated via the ΔΔCT method (Livak and Schmittgen, 2001), with *GAPDH* serving as the internal control (S6).

### 2.7 DR5::GFP distribution monitored by fluorescence

*DR5::GFP* plants were used to monitor the endogenous auxin response during DNSO. Either whole seedlings or 0.5 cm root segments (wounded roots) cut from 7-day-old seedlings were cultured on TSA-containing (3 μM) or TSA-free CIM. Half of the *DR5::GFP* seedlings or wounded roots were transferred to SIM, while the other half were left on CIM for further observation. Photographs were taken using a laser confocal microscope (Leica TCS SP8, Germany).

## 3 Results

### 3.1 TSA treatment at the callus induction stage promotes callus formation in wounded roots

**Figure 2:**
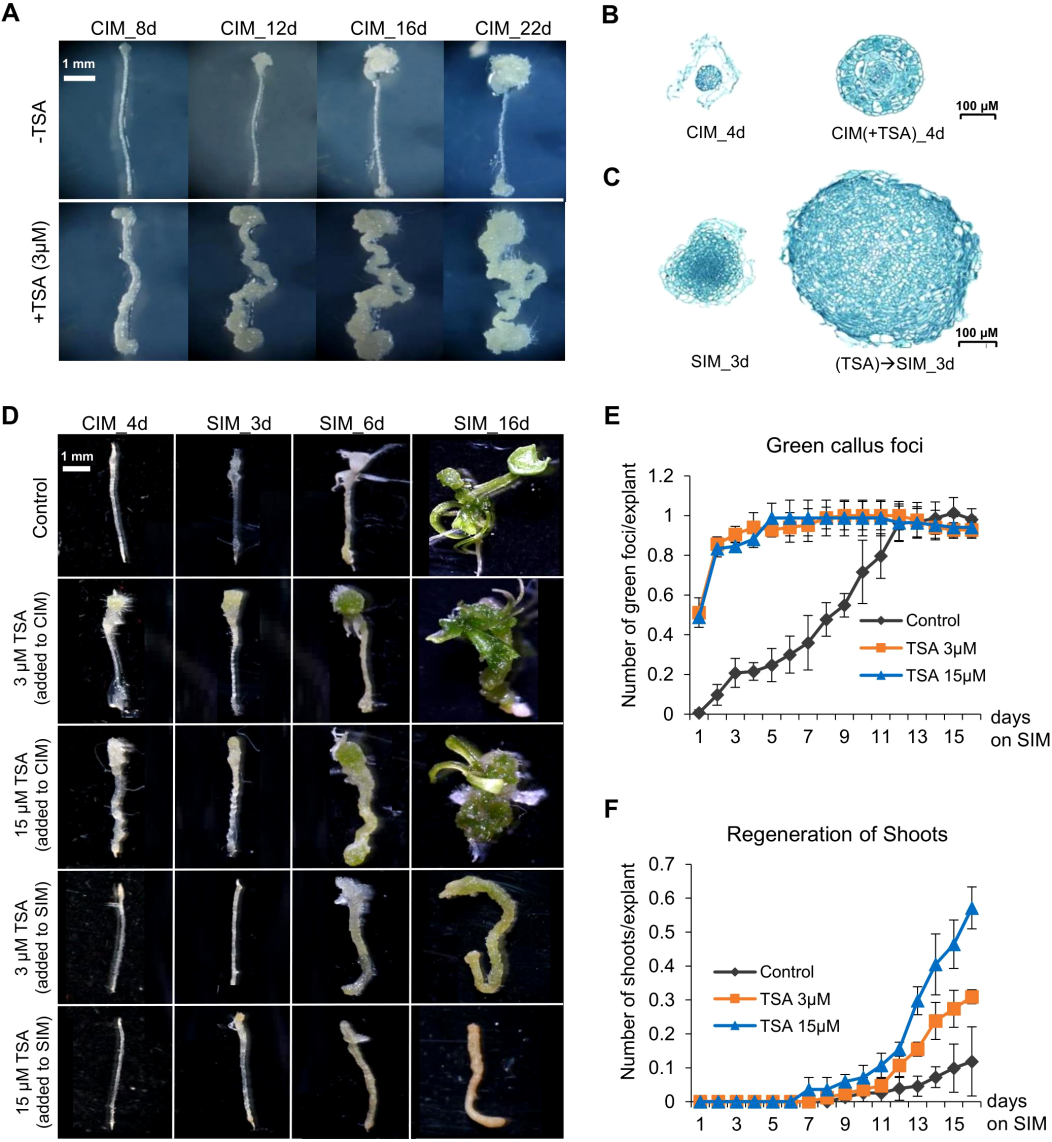
TSA treatment at the callus induction stage promotes callus formation and shoot regeneration from wounded roots. **(A)** TSA (3 μM) application in CIM not only promotes callus formation from the cut (wounded) ends but also initiates callus formation in the middle un-wounded regions, which is not observed in the wounded roots cultured on non-TSA containing CIM. **(B, C)** TSA treatment at the callus induction stage increases callus mass when cultured on both CIM and SIM. **(D)** TSA promotes shoot regeneration from wounded roots if added to CIM but inhibits shoot regeneration if added to SIM. **(E, F)** Time courses of regeneration of green foci and shoots from the wounded roots cultured on SIM following a 4-day callus induction with or without TSA. The number of green callus foci produced and the number of shoots generated were dramatically higher under TSA treatment than in the control. Error bars denote standard errors (n=3, biological replicates). CIM, callus induction; SIM, shoot induction.

To illustrate the role of histone acetylation marks in shoot organogenesis, we exploited the well-established root-based regeneration system in Arabidopsis and treated root segments with TSA, an inhibitor of Zn^2+^-dependent histone deacetylases (Grozinger and Schreiber, 2002; Gregoretti et al., 2004). Shoot formation was achieved following callus induction by culturing wounded roots in CIM before shoot regeneration was induced in the cytokinin-rich SIM (Skoog and Miller, 1957; Duclercq et al., 2011). The 0.5 cm long root segments assayed were named wounded roots in this study. We treated wounded roots in CIM supplemented with 3 μM TSA and monitored the process of callus production. The untreated control root segments produced calli at both ends of the excised wounded roots but not in the unwounded middle regions (**Figure 2A**). Compared with the controls at the same stage, the TSA-treated wounded roots produced larger calli (**Figures 2B, C**). Notably, callus production in non-TSA-treated root segments was limited to the ends of the explants, whereas callus formation in the TSA-treated root segments occurred in both the ends and the unwounded middle regions. These findings demonstrate that inhibiting HDAC by TSA not only promotes cell proliferation of wounded tissues at the cut ends but also activates callus production from the middle regions of the excised wounded roots.

### 3.2 TSA treatment at the callus induction stage increases the frequency of shoot organogenesis in wounded roots

We next examined the effect of TSA on shoot regeneration. The wounded roots were preincubated for 4 days in CIM supplemented with 0, 3 or 15 μM TSA and then transferred to SIM without TSA. First, TSA treatment accelerated the formation of green callus foci (centers of greenish callus masses indicative of shoot initiation, **Figures 2D, E**). The TSA-treated wounded roots showed green callus foci as early as day 1 on SIM, and all 150 wounded roots generated green callus foci (1 per root) by Day 5, whereas green callus foci were not generated from every control root until Day 11, although the total number of green callus foci was invariant between the control and the TSA-treated roots (**Figures 2D, E**). Furthermore, TSA treatment increased shoot regeneration (**Figures 2D, F**). The control wounded roots produced approximately 0.12 shoots per root after 16 days on SIM (**Figure 2F**). The shoot production of the roots treated with 3 μM and 15 μM TSA increased to 0.31 and 0.57 shoots per root, respectively, which was significantly greater than that of the control roots (*P* < 0.05, t test, 1F). In addition, we added 3 μM and 15 μM TSA to SIM, but no shoots were produced in either treatment (**Figure 2D**; **Supplementary Figure S2**). Our results indicate that the TSA-mediated promotion of shoot generation in wounded roots was stage dependent and effective only if it was applied at the callus induction stage.

### 3.3 TSA treatment at the callus induction stage activates genes involved in both callus and shoot formation in wounded roots

To understand the mechanisms underlying the TSA-mediated promotion of callus formation and shoot regeneration, we performed transcriptome analyses (**Figure 1A**). In the absence of TSA treatment, 6,499 and 5,649 DEGs were identified during the callus induction and shoot induction stages, respectively (**Figure 1B**; **Supplementary Figure S3**). These DEGs were categorized as “genes regulated during callus induction (CIRs)” (CIM_4d *vs.* wounded roots) and “genes regulated during shoot induction (SIRs)” (SIM_3d *vs.* CIM_4d). The upregulated CIRs were enriched in pathways essential for callus formation, such as the stem cell population or meristem maintenance, DNA replication and the cell cycle (**Supplementary Table S2**). The upregulated SIRs were enriched in pathways such as response to wounding, photosynthesis, cell wall biogenesis and modification (**Supplementary Table S3**).

In the presence of TSA treatment in CIM, 7,556 DEGs were identified in the wounded roots during callus formation (**Figure 1B**, CIR(T)s). Furthermore, compared with those in the control roots cultured on TSA-free CIM, 1,983 and 1,240 genes were up- and downregulated, respectively, in the wounded roots treated with TSA. (**Figure 1B**; **Supplementary Tables S12, 13**, “TSA-regulated”, CIM(+TSA) 4d *vs.* CIM_4d). However, there is a large number of genes remained unchanged in their expression (**Supplementary Table S14**). The 1,983 TSA-up genes were enriched in the processes of cell differentiation, response to hormones, cell wall metabolism, photosynthesis, etc. (**Supplementary Table S4**). Intriguingly, CIRs accounted for 11.5% of the TSA_up genes and 28.5% of the TSA_down genes, whereas SIRs accounted for 56.6% of the TSA_up genes and 31.5% of the TSA_down genes (**Figures 1C, D**). These findings indicate that inhibiting HDAC by TSA drastically shifts the transcriptome landscape in the wounded roots cultured on CIM, with preferential activation and repression of a significant number of genes that are otherwise regulated later at the shoot induction stage.

**Figure 3:**
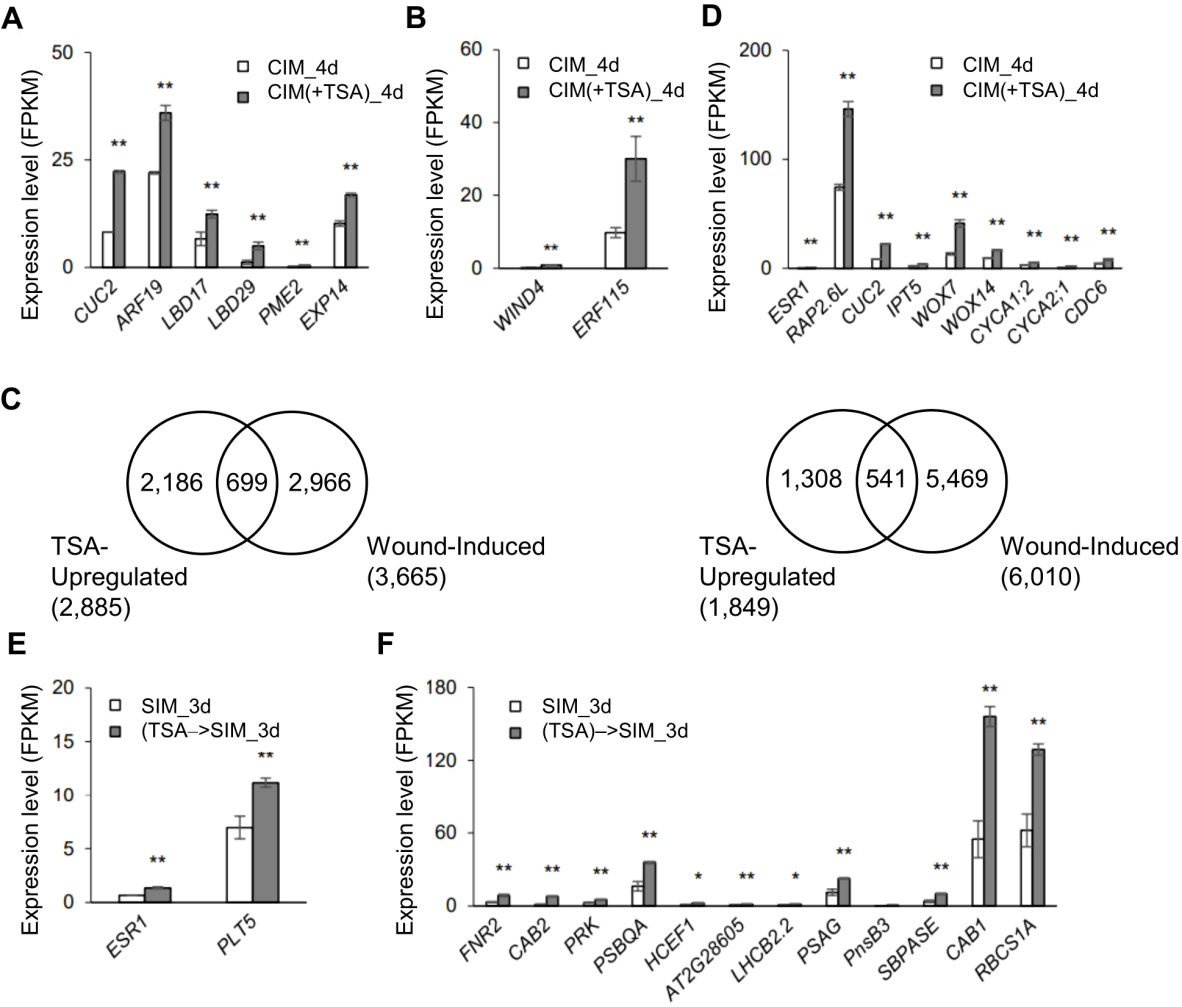
TSA treatment at the callus induction stage leads to upregulation of the genes involved in callus and shoot formation in wounded roots. **(A, B)** Expression levels of the callus formation-related genes in wounded roots upregulated by TSA at the callus induction stage (CIM(+TSA) 4d vs. CIM_4d), such as those involved in the *ARF-LBD* pathway **(A)** and wound signaling pathway **(B)**. **(C)** Venn diagrams showing substantial overlaps between the wound-regulated and TSA-regulated genes in wounded roots. TSA-up- and TSA-downregulated genes are those up- or downregulated in the TSA-treated wounded roots during callus formation (CIM(+TSA) 4d vs. CIM_4d). The wound-induced or wound-repressed genes are those up- or downregulated in wounded roots compared to unwounded roots (data from Rymen et al., 2019, fold change >1.5, P adj <0.001 as indicated in Rymen et al., 2019). **(D)** Expression levels of the shoot formation and cell differentiation-related genes in wounded roots that are upregulated by TSA at the callus induction stage (CIM(+TSA) 4d vs. CIM_4d). **(E, F)** Expression levels of the genes involved in shoot formation **(E)** and photosynthesis **(F)** in wounded roots at the shoot induction stage with TSA application in CIM ((TSA)->SIM_3d vs. SIM_3d). ** and * denote P adj <0.01 and P adj <0.05, respectively. Error bars denote standard errors (n=3, biological replicates).

Next, we examined the TSA-regulated genes (CIM(+TSA) 4d *vs.* CIM_4d) in detail and their relevance to the characterized callus formation pathways. In the absence of TSA, several auxin-induced callus formation pathway genes, such as *ARF19*, *LBD16*, *LBD17*, *LBD18*, and *E2Fa*, were upregulated 2.0- to 64.2-fold in CIM (**Supplementary Table S5**). In addition, several wound-induced callus formation pathway genes, such as *WIND3*, *ERF115* and *CYCD3;1*, were upregulated 2.4- to 15.1-fold (**Supplementary Table S5**). In the presence of TSA in CIM, the auxin-induced pathway genes *CUC2*, *ARF19*, *LBD17*, *LBD29*, *PME2* and *EXP14* were further upregulated by 1.7- to 4.4-fold (**Figure 3A**). The wound-induced pathway genes *WIND4* and *ERF115* were further upregulated 3.2- to 6.8-fold (**Figure 3B**). In addition, 24.2% of the TSA_up genes were upregulated, and 29.3% of the TSA_down genes were downregulated by tissue wounding (**Figure 3C**), demonstrating a substantial overlap of transcriptome reprogramming between TSA application and tissue wounding.

TSA application in CIM also upregulated a few genes involved in shoot regeneration, including *ESR1, RAP2.6L, CUC2, IPT5, WOX7* and *WOX14*, by 1.9- to 3.1-fold (**Figure 3D**). Notably, the *CUC1*, *2*, and *3* genes interact with *SHOOT MERISTEMLESS* (*STM*) during embryogenesis to establish, which further maintains the shoot stem cell niche of the shoot apical meristem (SAM) (Balkunde et al., 2017). These findings indicate that the *CUC* gene plays a crucial role in shoot generation. Moreover, empirical experiments involving T-DNA knockdown of the RAP2.6L gene revealed that it can regulate many key genes to play a key role in shoot development (Che et al., 2006). Furthermore, 75 of the TSA-up genes were annotated as related to “cell differentiation”. Seventy-one of the 75 genes were otherwise not upregulated during callus formation, including the genes encoding transcription factors, cyclin A proteins and cell wall organization factors (**Supplementary Table S6**). For example, the expression levels of *CYCA1;2*, *CYCA2;1* and *CDC6* (*CELL DIVISION CONTROL 6*) were also increased (3D). Hence, inhibiting HDAC by TSA at the callus induction stage promotes the regulatory networks that participate in callus formation and shoot induction in wounded roots.

### 3.4 TSA treatment at the callus induction stage leads to a sustained regulatory alteration in wounded roots when they are cultured on SIM

We then analyzed the transcriptomes of the wounded roots at the shoot induction stage following TSA application in CIM or SIM. Notably, in the presence of TSA on CIM, the wounded roots demonstrated a sustained regulatory alteration at the shoot induction stage when TSA was absent. A total of 221 genes presented increased expression, whereas 894 genes presented reduced transcription activity compared with the control wounded roots without TSA application (**Figure 1B**, (TSA)→SIM_3d *vs.* SIM_3d). The shoot regeneration genes *ESR1* and *PLT5* were upregulated 2.1- and 1.8-fold, respectively (**Figure 3E**). In addition, the 211 upregulated genes were enriched in photosynthesis-related pathways, such as *FERREDOXIN-NADP[+]-OXIDOREDUCTASE 2* (*FNR2*), *CHLOROPHYLL A/B BINDING PROTEIN 1 (CAB1*), *CAB2* and *PHOSPHORIBULOKINASE* (*PRK*) (**Figure 3F**), which was consistent with the faster appearance of green foci in response to TSA treatment.

In the presence of TSA in SIM, 867 genes were upregulated in the wounded roots compared with the control roots cultured on the TSA-free SIM (2B, SIM(+TSA) 3d *vs.* SIM_3d). Approximately two-thirds of those genes (561/867) were also upregulated by TSA during callus formation. However, in contrast to the enhancement of cell differentiation-related pathways by TSA during callus induction, few genes involved in cell differentiation were upregulated by TSA during shoot induction (**Supplementary Table S7**). Moreover, the 867 upregulated genes were enriched in toxin metabolism and detoxification processes, as well as interference with the metabolic balance of plant hormones (**Supplementary Table S7**), which was not observed when TSA was added to CIM since the enriched genes were involved mainly in cell development (**Supplementary Table S5**). These findings suggest that root tissues may be more susceptible to TSA during shoot induction. In summary, TSA application in CIM enhances differentiation-related pathways and shoot regeneration competence at different stages of development.

### 3.5 Upregulation of a set of genes by TSA in wounded roots is coupled with increased levels of H3K9/K14ac at gene loci

TSA targets Zn^2+^-dependent HDACs, which belong to the RPD3/HDA1 or HD-tuin type in Arabidopsis (Grozinger and Schreiber, 2002; Gregoretti et al., 2004). To determine whether the acetylation status of histones was altered after TSA application, the overall acetylation level of histone 3 (H3ac) in the TSA-treated wounded roots was probed. As expected, H3ac levels increased in the TSA-treated wounded roots relative to those in the untreated controls (**Supplementary Figure S4**). In addition, among the genes upregulated by TSA at the callus induction stage, 37.2% (1,072/2,885) were repressed by the histone acetyltransferase inhibitor γ-butyrolactone (MB3) (**Supplementary Figure S5**). These findings suggest that protein acetylation changes contribute to the transcriptional activation of these genes.

**Figure 4:**
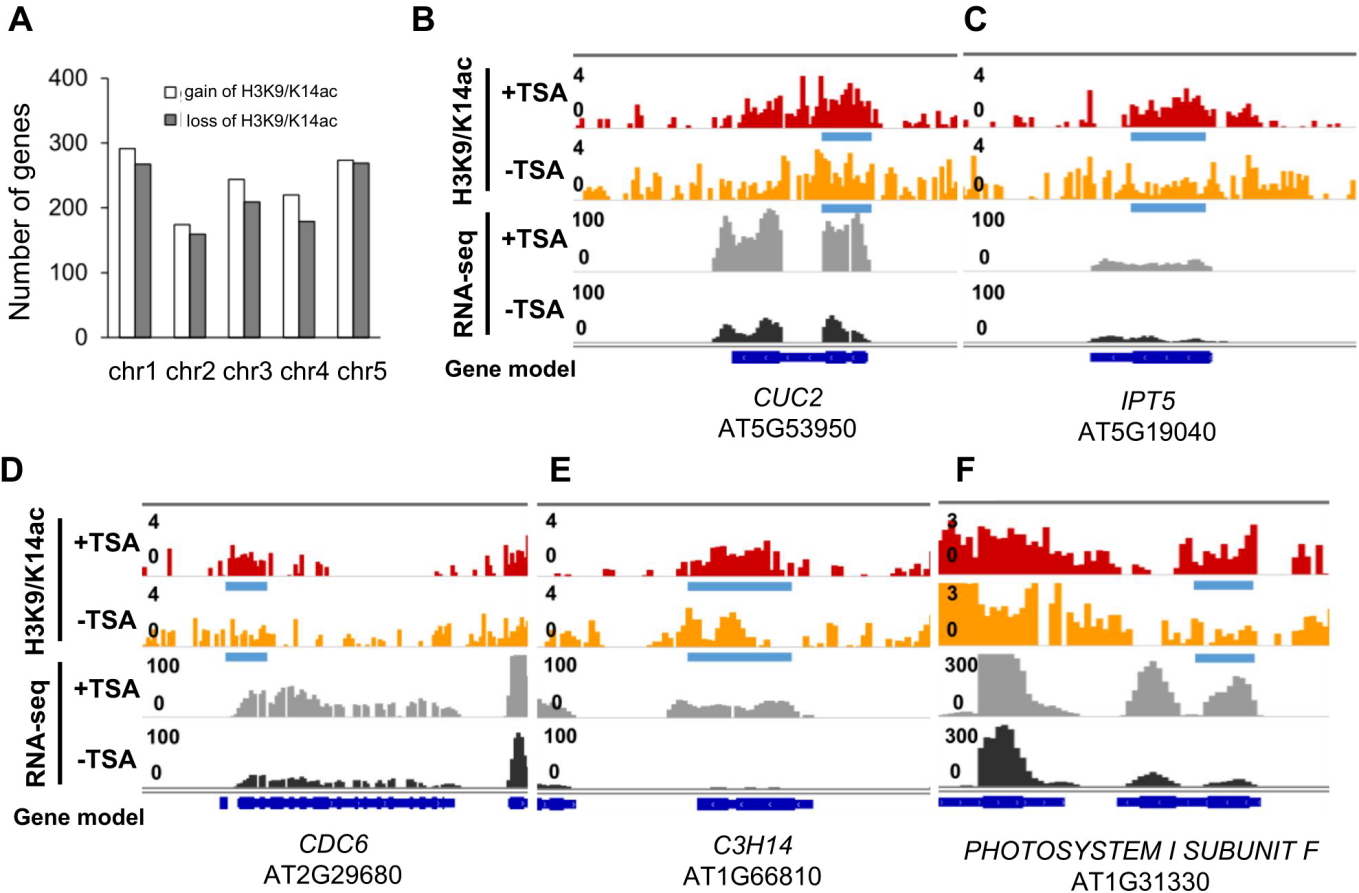
Upregulation of some shoot formation- and cell differentiation-related genes in the TSA-treated wounded roots are coupled with increased H3K9/K14ac enrichment levels by DiffBind analysis. **(A)** Bar graphs showing the number of genes with H3K9/K14ac gain or loss in the TSA-treated wounded roots during callus formation (CIM(+TSA) 4d vs. CIM_4d). **(B, C)** Browser shots show the association between increased H3K9/K14ac enrichment levels and expression levels for the genes involved in shoot formation, such as *CUC2* (1.13- and 2.79-fold) **(B)** and *IPT5* (1.12- and 1.88-fold) **(C)**. **(D–F)** Browser shots showing the association between increased H3K9/K14ac enrichment levels and expression levels for the genes involved in differentiation, such as *CDC6* for cell division control (2.55- and 1.89-fold) **(D)**, *C3H14* for cell wall biosynthesis (1.99- and 18.0-fold) **(E)**, and *PHOTOSYSTEM I SUBUNIT F* for photosynthesis (1.92- and 4.69-fold) **(F)**. Blue bars and red arrow denote differentially acetylated regions upon TSA treatment (CIM(+TSA) 4d vs. CIM_4d, P<0.05), identified by ChIP-seq data, which detailed information displayed in **Supplementary Table S12**.

To study the histone acetylation changes associated with TSA treatment in CIM, the genome-wide distribution of H3K9/K14ac was profiled via chromatin immunoprecipitation followed by sequencing (ChIP-seq, CIM(+TSA) 4d *vs.* CIM_4d). Genes with higher expression levels tended to have higher levels of H3K9/K14ac in both the TSA-treated and untreated wounded roots, as expected (**Supplementary Figure S6**). In total, 1,084 and 967 genes were associated with increases and decreases in H3K9/K14ac levels, respectively, in the wounded roots subjected to TSA treatment (**Figure 4A**; **Supplementary Table S8**). Our data demonstrate that the genome is subject to both gain and loss of histone acetylation upon TSA application, which is in accordance with previous reports (Rafehi et al., 2014; Anderson et al., 2017; Wang et al., 2021).

The 1,084 genes with H3K9/K14ac gains from TSA treatment were enriched in various pathways, including cell division, meristem development, shoot morphogenesis, cell wall organization or biogenesis, and response to light stimulus (**Supplementary Table S8**). These findings suggest that genes involved in the regulation of shoot formation are preferentially targeted by TSA. An investigation of the DEGs involved in DNSO revealed that the upregulation of *IPT5* by TSA was associated with increases in H3K9/K14ac levels in the gene sequences. The expression level of *CUC2* and *IPT5* was upregulated by 1.9-fold by TSA, which was associated with a differentially acetylated region across its gene body (**Figures 4B, C**). Furthermore, the upregulation of several differentiation-related genes, such as *CDC6*, which controls cell division; *C3H14*, which regulates cell wall biosynthesis; and *PHOTOSYNTHESIS II SUBUNIT F*, which is involved in photosynthesis, was also associated with increases in H3K9/K14ac levels (**Figures 4C–F**). In summary, our data suggest that the transcriptional regulation of *CUC2*, *IPT5*, *CDC6*, *C3H14*, and other differentiation-related genes was influenced by local H3K9/K14ac levels upon TSA treatment (**Figures 3**, **4**).

### 3.6 TSA circumvents tissue wounding to initiate shoot organogenesis in unwounded roots

**Figure 5:**
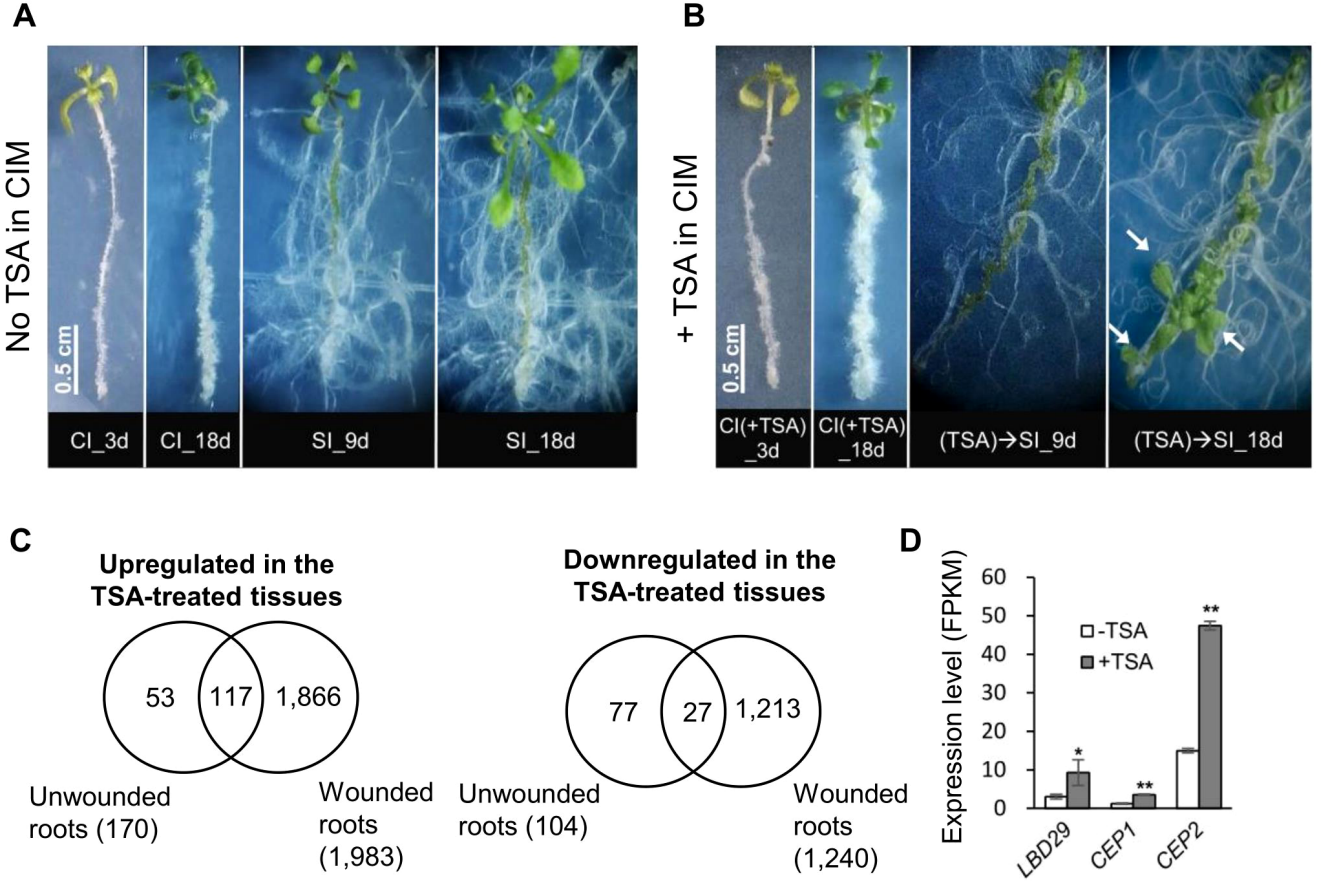
TSA treatment triggers shoot organogenesis along unwounded roots. **(A)** Shoots do not emerge from unwounded roots without TSA treatment. **(B)** Shoots appear along unwounded roots when pretreated with TSA. Arrows point to the regenerated shoots. Seedlings were cultured on CIM (with or without TSA) for 4 days and then transferred to SIM. **(C)** Venn diagrams show substantial overlaps between the TSA-regulated genes in wounded roots and in unwounded roots. Wounded roots and unwounded roots cultured on CIM (with or without TSA) for 4 days were collected and profiled. **(D)** *LBD29* in the *LBD-ARF* callus formation pathway, *CEP1* and *CEP2* involved in lateral root growth is upregulated by TSA in unwounded roots. * and ** denote P adj <0.05 and P adj <0.01, respectively. Error bars denote standard errors (n=3, biological replicates). CIM, callus induction; SIM, shoot induction.

Previous studies have demonstrated that wound signaling is a prerequisite for shoot organogenesis in conventional tissue culture (Iwase et al., 2015). We observed that TSA treat initiated callus production in the unwounded tissues of root segments that were otherwise unable to produce calli (**Figure 2A**). This finding prompted us to examine whether TSA treatment could also trigger shoot regeneration from unwounded roots. Seedlings were preincubated on CIM with or without TSA for 4 days before being transferred to TSA-free SIM. In the absence of TSA treatment, no shoot regeneration was observed in unwounded roots after 20 days on SIM (**Figure 5A**). However, in the presence of TSA, shoot regeneration occurred in 28 of 284 unwounded roots (**Figure 5B**). Notably, TSA treatment was able to circumvent tissue wounding to trigger shoot regeneration in unwounded roots.

To explore the genes regulated by TSA, transcriptome data from unwounded roots collected from seedlings incubated on CIM (for 4 days) with or without TSA were profiled. In total, 177 and 110 genes were up- and downregulated by TSA, respectively, in unwounded roots (**Figure 5C**). Pathway analyses revealed that the 177 upregulated genes were enriched in processes related to secretory vesicles and cell walls, whereas the 110 downregulated genes were enriched in processes related to amino acid exports, responses to temperature stimulus, etc. Furthermore, 68.9% (117/170) and 26.0% (27/104) of the TSA-regulated genes in unwounded roots were up- and downregulated, respectively, by TSA in wounded roots (**Figure 5C**). Upon TSA treatment of unwounded roots, *LBD29* was upregulated 3.1-fold (**Figure 5D**; **Supplementary Table S9**). Expression of the genes encoding *CYSTEINE ENDOPEPTIDASE* 1 and 2 (*CEP1* and *CEP2*), which participate in cellulose wall degradation, was elevated by 2.9- and 3.2-fold, respectively (5D; **Supplementary Table S9**). The expression levels of *LBD29*, *CEP1* and *CEP2* were also elevated by 1.9- to 4.4-fold by TSA in wounded roots (**Supplementary Table S5**), indicating their transcriptional regulation by TSA in both wounded and unwounded roots. Notably, none of the wound signaling pathway genes were significantly changed by TSA in unwounded roots (**Supplementary Table S9**). Thus, our data demonstrate that TSA potentially triggers shoot organogenesis in unwounded roots in an *ARF-LBD*-associated manner, which bypasses wound signaling in this case.

### 3.7 TSA application at the callus induction stage downregulates the genes involved in auxin biosynthesis in wounded roots

**Figure 6:**
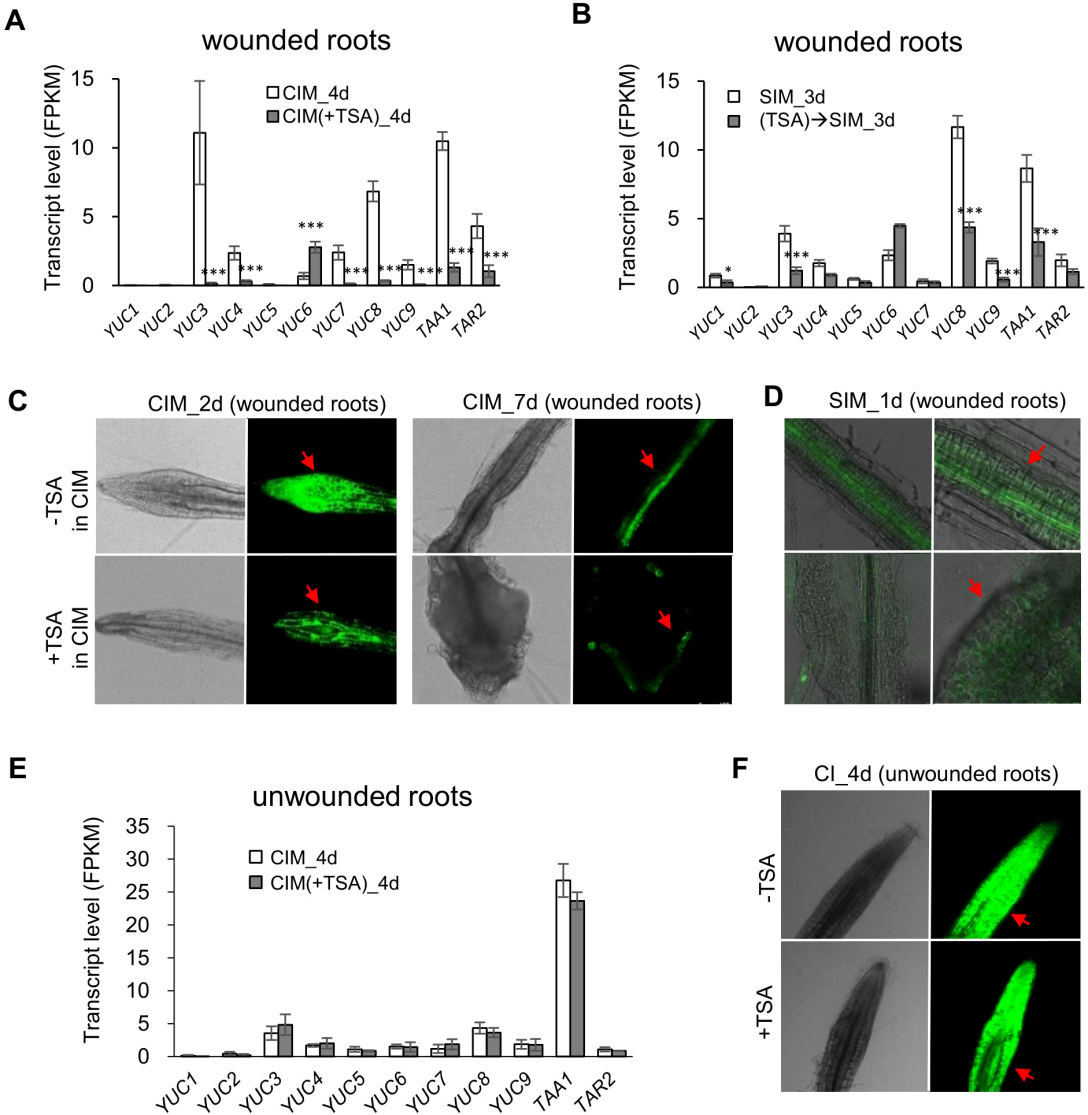
TSA treatment downregulates the expression levels of auxin biosynthetic genes and reduces auxin contents in wounded roots while not impacting auxin biosynthetic genes or auxin content in unwounded roots. **(A)** Expression of auxin biosynthetic genes is downregulated in wounded roots at the callus induction stage by application of TSA (3 μM) in CIM. **(B)** Expression of auxin biosynthetic genes is downregulated during the shoot regeneration stage in the wounded roots pretreated by TSA. **(C, D)** TSA (3 μM) in CIM reduces the *DR5::GFP* reporter signal in wounded roots both at the callus induction stage and at the shoot induction stage. **(E)** TSA does not impact the expression levels of auxin biosynthetic genes in unwounded roots. **(F)** TSA does not influence the *DR5::GFP* reporter signal in unwounded roots. * and ** denote P adj <0.05 and P adj <0.01, respectively. Error bars denote standard errors (n=3, biological replicates). CIM, callus induction; SIM, shoot induction. Photographs were taken with a laser confocal microscope (Leica TCS SP8, Germany).

Plant hormones play a key role in the DNSO system. Our transcriptome data demonstrated that the TSA-suppressed genes were enriched in several hormone biosynthetic processes (*e.g.*, auxin and gibberellin biosynthesis) and in hormone responsiveness (*e.g.*, the auxin responsive *GH3* family) (**Supplementary Table S3**). In particular, eight auxin biosynthetic genes, including six *YUCCA* genes and two *TAA* genes, were significantly downregulated by 4.0- to 84.7-fold upon TSA at the callus induction stage (**Figure 6A**; **Supplementary Table S10**). Furthermore, the wounded roots cultured on SIM following preincubation on CIM containing TSA presented decreased expression levels of auxin biosynthesis pathway genes (**Figure 6B**, (TSA)→SIM_3d *vs.* SIM_3d).

The reduction in auxin biosynthesis in the wounded roots caused by the presence of TSA on CIM was further confirmed by an auxin-sensitive *DR5* promoter-driven GFP (*DR5::GFP*) reporter that was introduced into the plants. In the absence of TSA treatment, the *DR5::GFP* reporter signal was detected throughout the wounded roots on Day 2 and then faded and was mainly observed in pericycle cells on Day 7 (CIM_2d and CIM_7d in **Figure 6C**). Upon TSA treatment, the *DR5::GFP* reporter signal significantly decreased in the wounded roots on Day 2 (**Figure 6C**). On Day 7, the *DR5::GFP* reporter signal was located on the surface of the enlarged calli in the TSA-treated wounded roots (**Figure 6C**), which was similar to the auxin-responsive pattern before shoot initiation (Meng et al., 2017). These changes in the auxin signal distribution caused by TSA treatment were also observed in the wounded roots after they were transferred to SIM (**Figure 6D**), indicating that the TSA-mediated reduction in auxin levels and the change in auxin distribution contributed to the promotion of shoot formation. On the other hand, neither the expression levels of auxin biosynthetic genes nor the *DR5::GFP* reporter signal significantly differed between the TSA-treated and untreated unwounded roots (**Figures 6E, F**), indicating that the effect of TSA on auxin biosynthesis and redistribution was also dependent on tissue wounding.

## 4 Discussion

**Figure 7:**
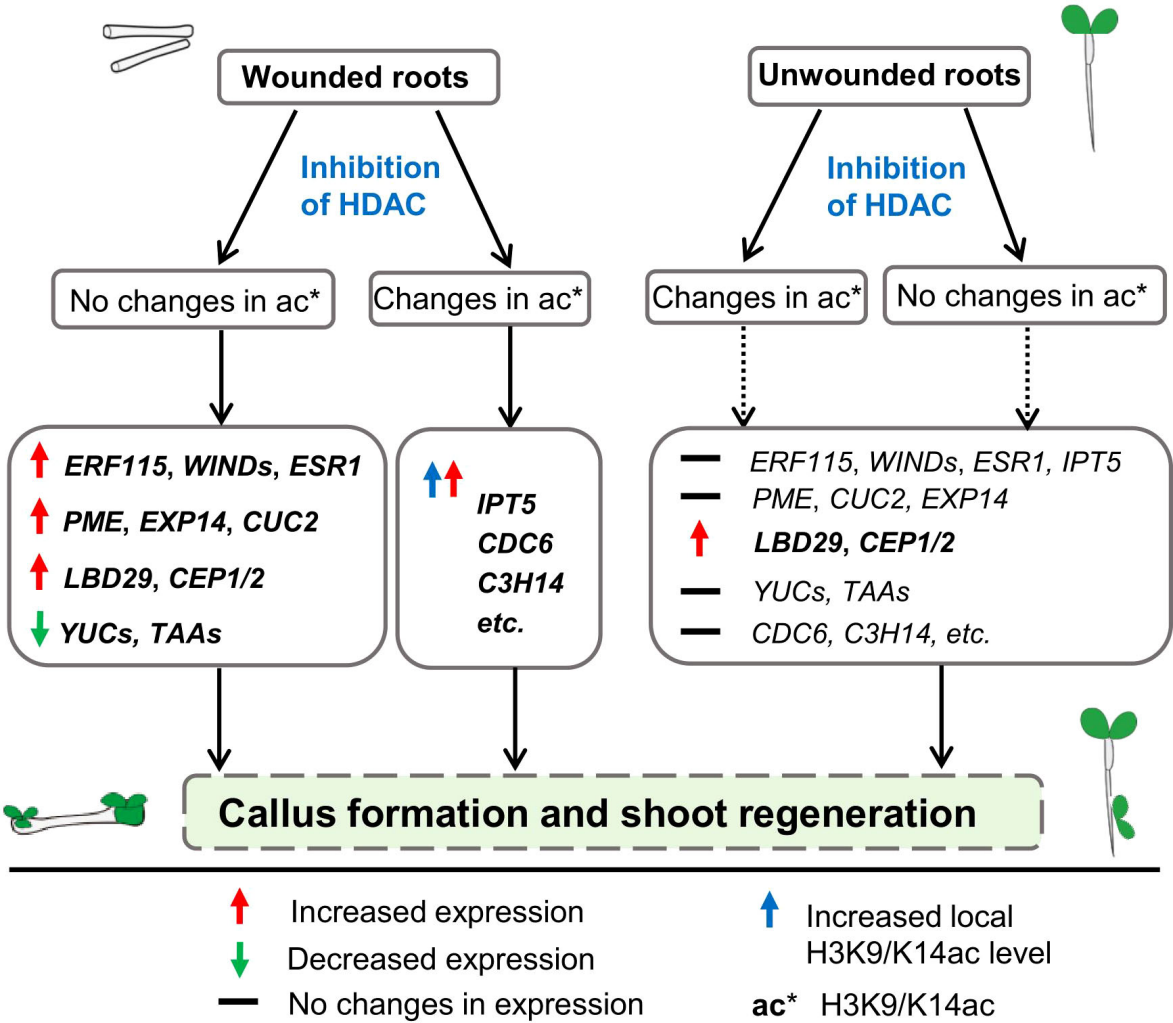
A proposed model for the involvement of histone deacetylases in *de novo* shoot organogenesis. The proposed model is based on our results that inhibition of HDAC by TSA at the callus induction stage is able to promote shoot regeneration in wounded roots and circumvent tissue wounding to initiate shoot regeneration in unwounded roots. The HDAC inhibition-mediated promotion of callus and shoot regeneration in wounded roots is associated with concomitant increased expression of wound signaling (e.g., *ERF115*, *WIND4*, *ESR1*) and ARF-LBD (e.g., *LBD29*, *PME2*, *CUC2*, *EXP14*) pathway genes and decreased expression of auxin biosynthetic genes (e.g., *YUCCAs*, *TAAs*). On the other hand, the activation of shoot organogenesis from unwounded roots is associated with increased expression of the ARF-LBD pathway gene *LBD29*, which bypasses wound signaling and auxin biosynthetic genes. Upregulation of some shoot formation-associated genes such as *IPT5* and some differentiation-related genes by inhibiting HDAC coupled with increased local H3K9/K14ac levels demonstrates the involvement of histone acetylation in shoot regeneration.

Plants reprogram somatic cells to regenerate new tissues and organs following injury or hormonal cues. In this study, we demonstrated that the inhibition of histone deacetylases (HDAC) promotes callus and shoot formation from wounded roots and circumvents tissue wounding to activate shoot regeneration in unwounded roots (**Figure 7**). This HDAC inhibition-mediated promotion of shoot organogenesis in wounded roots is associated with concomitant increases in wound signaling and *ARF-LBD* pathways and decreases in auxin biosynthesis. On the other hand, the activation of shoot organogenesis from unwounded roots is associated with an increase in the *ARF-LBD* pathway, which bypasses wound signaling and auxin biosynthetic genes. We also provide evidence that the inhibition of HDACs regulates the transcriptional apparatus of genes relevant for shoot formation via both local histone acetylation-dependent and acetylation-independent mechanisms.

### 4.1 HDAC inhibition augments wounding signaling for shoot organogenesis in a wound-dependent manner

Accumulating evidence suggests that a wounding signal is indispensable for *de novo* shoot organogenesis from roots in the conventional tissue culture of *Arabidopsis thaliana* (Iwase et al., 2015; Ikeuchi et al., 2019). A recent study revealed that a majority of the genes rapidly induced by wounding are characterized by histone acetylation before and/or shortly after wounding in Arabidopsis roots and that inhibition of GNAT-MYST histone acetyltransferases strongly blocks wound-induced transcriptional activation (Rymen et al., 2019). Our data demonstrate that in the absence of wounding (unwounded roots), inhibiting HDACs at the callus induction stage leads to initiation of shoot organogenesis (**Figure 5A**). In the presence of wounding (wounded roots), inhibiting HDAC at the callus induction stage leads to increased expression levels of the wound signaling genes involved in shoot organogenesis (**Figures 2** and **3**). Interestingly, in TSA-treated unwounded roots, expression of the *ARF-LBD* pathway increased, whereas that of the wounding pathway remained unchanged. These results imply that HDAC inhibition is more likely to circumvent rather than substitute for tissue wounding to initiate shoot regeneration in unwounded roots and that the promotional effect of TSA on wound signaling in wounded roots occurs downstream of tissue wounding. Callus and shoot formation is a complex genetic trait that is controlled by various endogenous and environmental cues. In addition, epigenetic regulation plays a pivotal role throughout shoot regeneration, although the precise mechanism remains to be fully elucidated. A previous study revealed that the auxin response factor *ARF* can activate the transcription factor *LATERAL ORGAN BOUNDARIES DOMAIN* (*LBD*), which is associated with shoot formation. However, many empirical experiments have confirmed that both *LBD* and *ARF* mutations affect shoot formation in both single and double mutants. Thus, TSA treatment of unwounded roots induced shoot generation, although the *LBD-* and *ARF*-related genes presented similar expression trends as those in the wounded roots did, indicating that there are unknown pathways involved in shoot formation that need further study (Okushima et al., 2007; Lee et al., 2009; Berckmans et al., 2011).

### 4.2 Involvement of histone deacetylases in shoot organogenesis

Previous studies have shown that inhibition of the GNAT-MYST group of histone acetyltransferases strongly blocks callus formation at wound sites, which is consistent with the promotional effect of the inhibition of histone deacetylases in our assays. Furthermore, our data demonstrate that the inhibition of HDAC by TSA at the callus induction stage, but not at the shoot induction stage, promotes callus proliferation and ensures shoot regeneration in wounded roots (**Figure 2**). The genes upregulated by TSA during callus formation are enriched in cell differentiation-related pathways, whereas few DNSO genes are upregulated by TSA during shoot induction (**Supplementary Tables S3, S6**). In contrast, few genes involved in cell differentiation were upregulated when TSA was added to SIM, and genes involved in toxin metabolism and detoxification processes were upregulated instead, which supports a stage-specific role of TSA in shoot organogenesis. We know that histone acetylation is one of the most significant chromatin modifications in eukaryotes and is typically mediated by the opposite functions of HATs and HDACs, the latter of which are thought to be components of multiprotein complexes that include transcriptional repressors, scaffold proteins, and other cofactors.

According to a prior study, RNAi-mediated silencing of *HDAC* (HDACi) genes leads to the enrichment of acetylated histones and nonhistone proteins that are important for controlling the expression of genes, enzymatic activity, and cell division. These findings indicate that TSA may have an impact on chemical and enzymatic processes in addition to histone acetylation and the acetylation of nonhistone proteins and ultimately may affect various developmental processes. Researchers have reported that HDA6 is associated with heterochromatin formation, gene repression, stress tolerance, flowering time, and circadian rhythm; in addition, HDA6 deficiency can also lead to significant increases in H3K9ac and H3K14ac and gene activation (Lin et al., 2020). Thus, as evidenced by the HDACi of TSA-mediated potential associations with different factors or developmental stages, the mechanism through which TSA is involved in regulating the formation of calli and shoots is complex and needs to be clarified in further studies (Zhang et al., 2013; Wei et al., 2022).

In wounded roots, 1,084 genes, including *IPT5*, which promotes shoot formation, are associated with increases in H3K9/K14ac levels (4). In addition, some genes related to cell differentiation, such as *CDC6*, which is involved in cell division control; *C3H14*, which is important for cell wall biosynthesis; and *PHOTOSYSTEMSUBMIT F*, presented increased H3K9/K14ac levels in wounded roots upon TSA treatment (**Figure 4**). These findings suggest that the transcription status of these shoot formation- and cell differentiation-related genes is affected by the local histone acetylation level after TSA application.

Notably, 967 genes were associated with decreases in H3K9/K14ac levels in the wounded roots subjected to TSA treatment. Moreover, many of the DNSO-related genes whose expression was upregulated by TSA application were not associated with H3K9/K14ac changes in the wounded roots. Studies in animals have demonstrated that the genome is subject to both acetylation and deacetylation upon TSA application and that changes in gene expression are not always associated with histone acetylation (Rafehi et al., 2014; Anderson et al., 2017; Wang et al., 2021). Histone deacetylases are in fact a supergene family; their organelle location specificity and functional specificity are important in many plant development processes. Therefore, the use of nonspecific TSA inhibits HDACs leads to uncertain results and may also potentially reveal why adding TSA can promote callus formation in the CIM stage but inhibit shoot generation in the SIM stage (Demetriou et al., 2010; Fong et al., 2006; Probst et al., 2004). Moreover, changes in the distribution of histone modifications other than acetylation, such as H3K4me3, may lead to gene transcriptional regulation after TSA treatment (Rafehi et al., 2014), which may further decrease the significance of the correlation between transcription and acetylation.

Thus, we propose that HDAC inhibition promotes shoot regeneration in both histone acetylation-dependent and histone acetylation-independent manners. Inhibiting HDACs regulates the expression levels of shoot organogenesis-related genes either directly through modification of the local histone acetylation levels or indirectly through influence of the expression levels of upstream genes (**Figure 7**). Given that HDACs target both histones and nonhistone proteins, some unknown nonhistone proteins modified by HDACs may also contribute to shifts in the transcriptome.

### 4.3 Inhibition of HDACs reduces auxin biosynthesis in wounded roots

Temporal modulation of hormones plays a key role in *de novo* shoot organogenesis. As indicated by the response of *DR5::GFP* reporter expression, auxin levels peak at approximately 2-3 days on CIM and then decline over time in growing calli (Gordon et al., 2007). Our data demonstrated that upon TSA treatment in wounded roots, although the expression levels of ARF-LBD pathway genes downstream of auxin were increased, the *DR5::GFP* reporter expression level was decreased, which was associated with reduced expression levels of auxin biosynthetic genes (**Figures 6A–D**). Hence, our results suggest that a lower auxin content resulting from a reduction in auxin biosynthesis contributes to the promotion of shoot organogenesis by TSA in wounded roots. On the other hand, neither the expression of auxin biosynthetic genes nor the *DR5::GFP* reporter expression differed in unwounded roots (**Figures 6E, F**), indicating that the reduction in auxin biosynthesis by TSA also occurred downstream of tissue wounding.

A recent study revealed that TSA promotes somatic embryogenesis on the adaxial side of cotyledons (Wojcikowska et al., 2018). These authors reported an increase in auxin biosynthesis in response to TSA treatment, which contradicts the decrease in auxin content observed in our assays. The process of pluripotency acquisition by somatic cells includes the silencing of genes to erase original tissue memory and the priming of additional cell type specification genes, both of which are cell type specific. The different effects of TSA on auxin biosynthesis in cotyledons and wounded roots may reflect different transcriptional regulation and cell identity in various tissues.

## 5 Conclusions

*In vitro* plant regeneration through *de novo* shoot organogenesis has been the subject of intensive investigations over the last several decades because of its significance in fundamental research and applications in genetic engineering. In this study, we show that inhibiting HDACs via the exogenous application of inhibitors can upregulate the wound signaling and ARF-LBD pathways and downregulate auxin biosynthesis to promote shoot organogenesis in wounded roots of *Arabidopsis thaliana*. Moreover, inhibiting HDACs increases the *ARF-LBD* pathway and circumvents wound signaling to initiate shoot organogenesis from unwounded roots. Our investigation provides important insights into the genetically and epigenetically controlled balance in *de novo* shoot organogenesis.

## Data Availability

The datasets presented in this study can be found in online repositories. The names of the repository/repositories and accession number(s) can be found in the article/**Supplementary Material**.

## Supplementary material

Supplementary Figure 1 Verification for the consistency between the transcriptome and qPCR results. Gene expression was normalized to GAPDH. Error bars denote standard errors (n=3, biological replicates).

Supplementary Figure 2 Time courses of shoot regeneration of green foci from the wounded roots cultured on SIM (with or without TSA) following a 4-day callus induction on CIM. Error bars denote standard errors.

Supplementary Figure 3 Heatmaps demonstrating expression patterns of CIRs and SIRs. **(A)** Expression patterns of the upregulated CIRs. **(B)** Expression patterns of the downregulated CIRs. **(C)** Expression patterns of the upregulated SIRs. **(D)** Expression patterns of the downregulated SIRs. CIR, DEGs during callus induction (CIM_4d vs. wounded roots); SIR, DEGs during shoot induction (SIM_3d vs. CIM_4d).

Supplementary Figure 4 Immunoblot of the total and acetylated (Ac) histone H3 in the wounded roots cultured on CIM for 4 days with or without TSA indicating that TSA treatment increases H3ac levels in wounded roots.

Supplementary Figure 5 Venn diagram showing substantial overlap between the MB3 (an acetyltransferase inhibitor)-repressed genes and the TSA-enhanced genes in the wounded roots. The genes upregulated in the TSA-treated wounded roots are defined as (CIM (+TSA) 4d vs. CIM_4d, Padj<0.001, FC>1.5).

Supplementary Figure 6 Positive correlation between the enrichment levels of H3K9/K14ac and the transcription levels in the ChIP-seq experiments in the TSA-untreated **(A, B)** and -treated wounded roots **(C, D)**. The genes with FPKM>0 were evenly classified into five groups, which were profiled together with the silent genes (FPKM=0). The analyzed sequences included the 3k bp upstream of TSS, 3k bp downstream of TES, and the rest of the gene body.
