# Supplementary material for: Inhibition of histone deacetylase in Arabidopsis root calli promotes de novo shoot organogenesis
Source: Front Plant Sci. 2025 Jan 27;15:1500573. doi: 10.3389/fpls.2024.1500573 (PMC11807735; doi:10.3389/fpls.2024.1500573)

Supplemental Figure S1

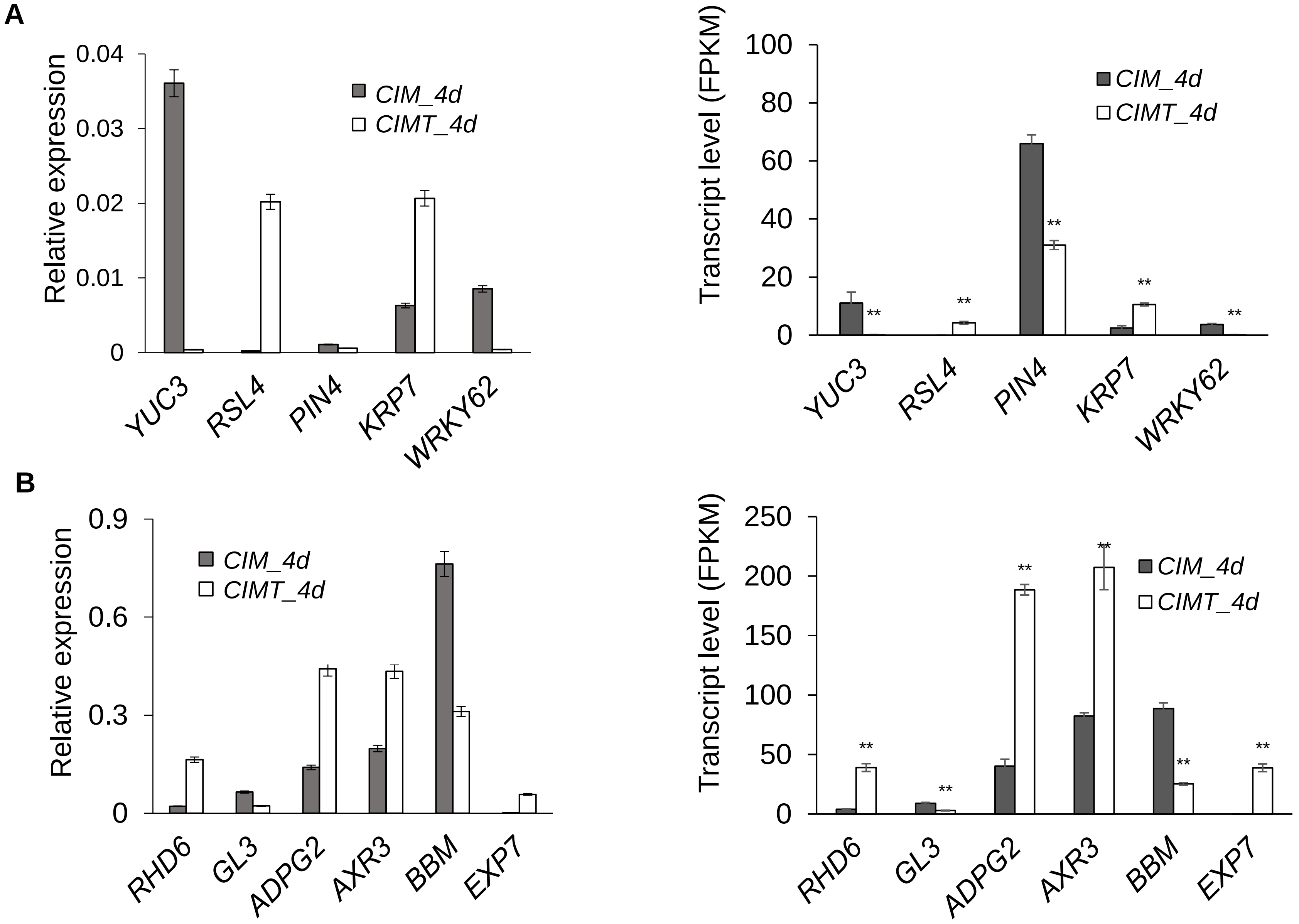

Supplemental Figure S2

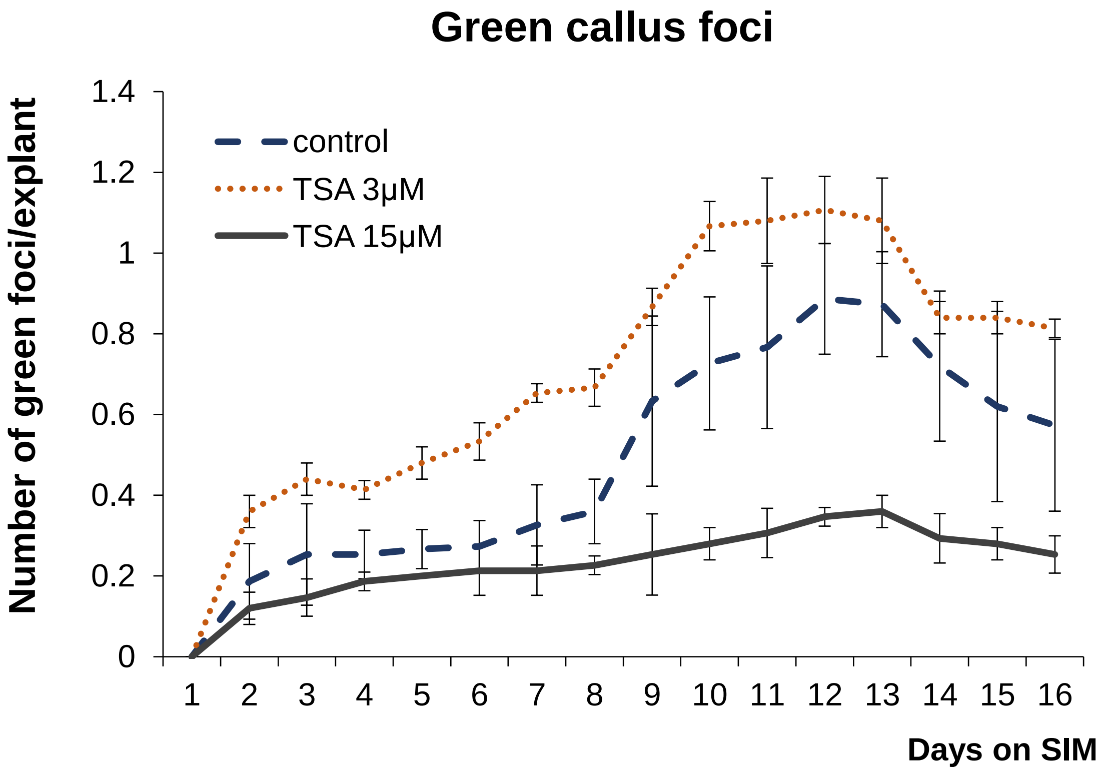

Supplemental Figure S3

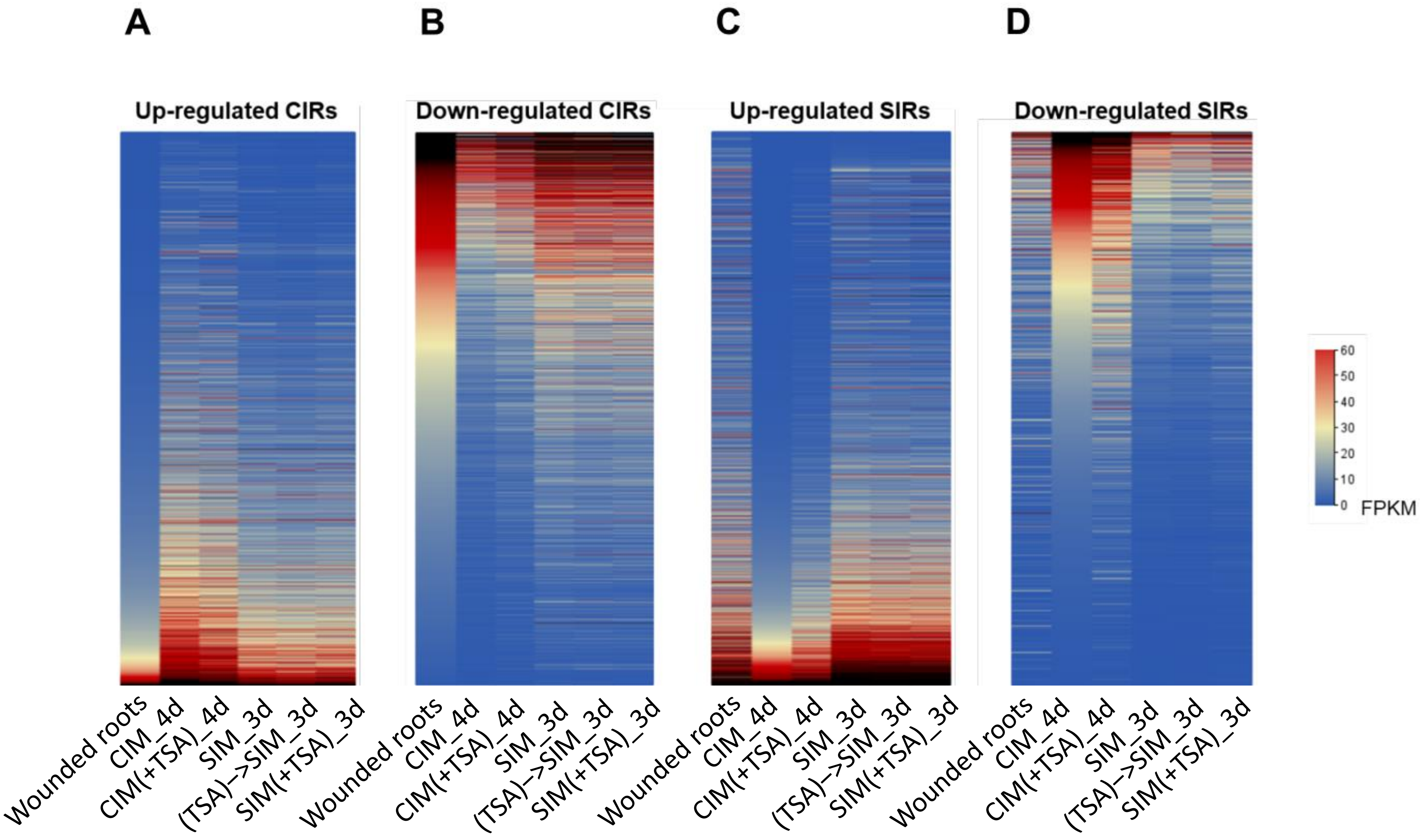

Supplemental Figure S4

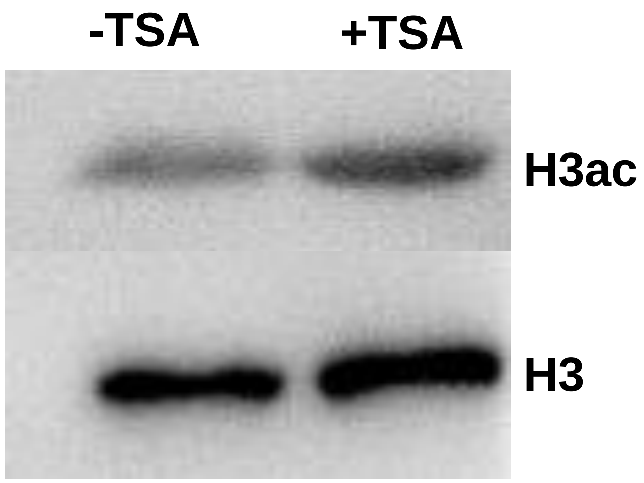

Supplemental Figure S5

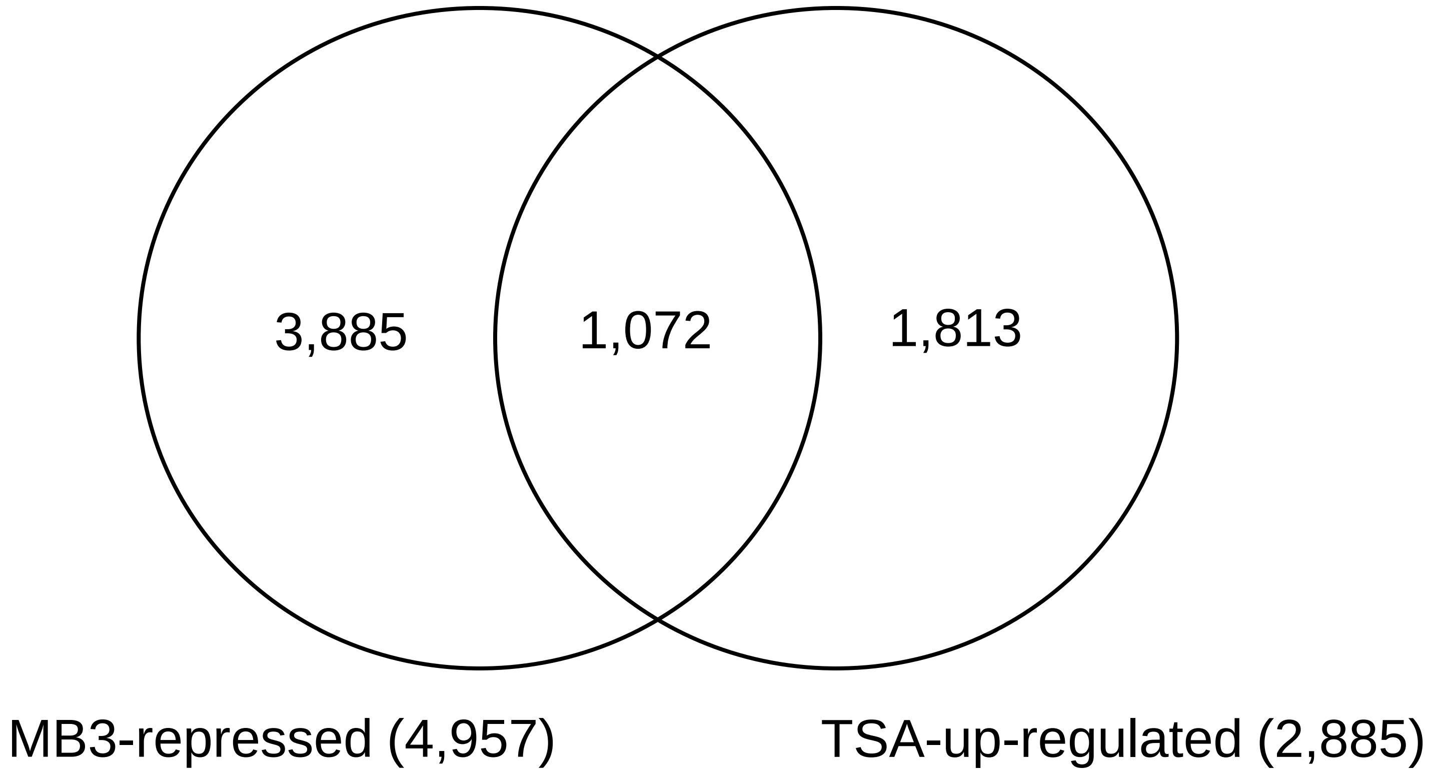

Supplemental Figure S6

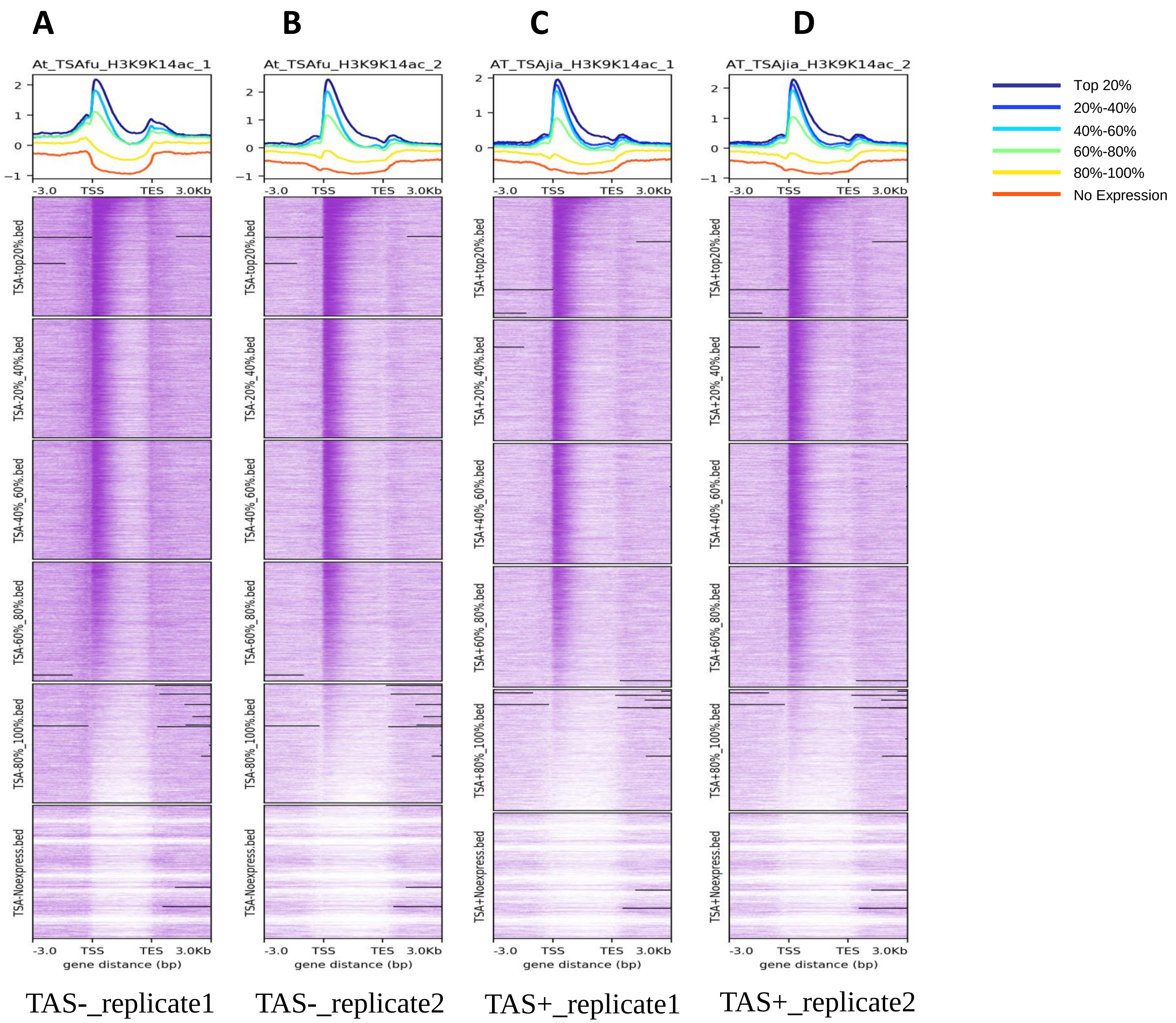

Supplement: Supplementary Figure 1 — Verification for the consistency between the transcriptome and qPCR results. Gene expression was normalized to GAPDH. Error bars denote standard errors (n=3, biological replicates). [file DataSheet1.pdf]
